# Fatal Myelotoxicity Following Palliative Chemotherapy With Cisplatin and Gemcitabine in a Patient With Stage IV Cholangiocarcinoma Linked to Post Mortem Diagnosis of Fanconi Anemia

**DOI:** 10.3389/fonc.2019.00420

**Published:** 2019-05-22

**Authors:** Nils W. Engel, Simon Schliffke, Ulrich Schüller, Christian Frenzel, Carsten Bokemeyer, Christian Kubisch, Davor Lessel

**Affiliations:** ^1^Department of Oncology, Haematology and Bone Marrow Transplantation with Section Pneumology, Hubertus Wald Tumorzentrum, University Medical Center Hamburg-Eppendorf, Hamburg, Germany; ^2^Institute of Neuropathology, University Medical Center Hamburg-Eppendorf, Hamburg, Germany; ^3^Department of Pediatric Hematology and Oncology, University Medical Center, Hamburg-Eppendorf, Germany; ^4^Research Institute Children's Cancer Center Hamburg, Hamburg, Germany; ^5^Institute of Human Genetics, University Medical Center Hamburg-Eppendorf, Hamburg, Germany

**Keywords:** fanconi anemia, FANCA, cholangiocarcinoma, genomic instability, myelotoxicity

## Abstract

Unrecognized genome instability syndromes can potentially impede the rational treatment of cancer in rare patients. Identification of cancer patients with a hereditary condition is a compelling necessity for oncologists, giving varying hypersensitivities to various chemotherapeutic agents or radiation, depending on the underlying genetic cause. Omission of genetic testing in the setting of an overlooked hereditary syndrome may lead to unexpected and unbearable toxicity from oncological standard approaches. We present a case of a 33-year-old man with an early-onset stage IV intrahepatic cholangiocarcinoma, who experienced unusual bone marrow failure and neutropenic fever syndrome as a consequence of palliative chemotherapy containing cisplatin and gemcitabine, leading to a fatal outcome on day 25 of his first chemotherapeutic cycle. The constellation of bone marrow failure after exposure to the platinum-based agent cisplatin, the presence of an early-onset solid malignancy and the critical appraisal of further phenotypical features raised suspicion of a hereditary genome instability syndrome. Whole-exome sequencing from buccal swab DNA enabled the post mortem diagnosis of Fanconi anemia, most likely linked to the fatal outcome due to utilization of the DNA crosslinking agent cisplatin. The patient's phenotype was exceptional, as he never displayed significant hematologic abnormalities, which is the hallmark of Fanconi anemia. As such, this case stresses the importance to at least question the possibility of a hereditary basis in cases of relatively early-onset malignancy before defining an oncological treatment strategy.

## Introduction

Genome instability syndromes are a group of inherited conditions caused by germline mutations in genes encoding DNA repair proteins involved in diverse DNA damage response (DDR) pathways, resulting in defects in genome maintenance, sensitivity toward various genotoxic agents, and early-onset cancer susceptibility (1, 2). Besides the increased incidence of various cancers, based on the underlying genetic cause, major symptoms of genome instability syndromes include developmental and craniofacial abnormalities, neurological deficiencies, immunodeficiency, cardiovascular diseases, metabolic abnormalities, and clinical signs of premature/accelerated aging. The phenotypic spectrum of these disorders and especially the hypersensitivity toward genotoxic agents, many of which are actually used in antineoplastic therapy, result from the mutation-induced and thereby gene-specific defects in different DDR pathways as each recognizes and/or repairs a highly specific class of DNA lesions (3). Known genome instability syndromes are, for example, caused by mutations in genes encoding DNA helicases like *WRN* in Werner Syndrome and *BLM* in Bloom syndrome, mutations in genes involved in nucleotide excision repair like *XPA-HPV* in Xeroderma pigmentosum and *CSA* and *CSB* in Cockayne syndrome, mutations in genes involved in DDR in response to the DNA double-strand breaks like *ATM* in Ataxia-telangiectasia and *NBS1* in Nijmegen breakage syndrome, mutations in genes involved in protein-DNA crosslink repair like *SPRTN* in Ruijs-Aalfs syndrome, and mutations in genes involved in interstrand DNA crosslink repair like *FANCA-FANCU* in Fanconi anemia (1, 2).

Fanconi anemia (FA) is a multisystem disorder characterized by physical anomalies, hematologic abnormalities including progressive bone marrow failure and pancytopenia, early-onset leukemia and solid cancer susceptibility, as well as hypersensitivity toward DNA crosslinking agents such as mitomycin C, cisplatin and diepoxybutane (4, 5). The majority of patients have normal blood counts at birth, and develop hematological abnormalities including anemia, neutropenia, thrombocytopenia, and even pancytopenia within the first decade of life. Physical anomalies are present in around the half of affected individuals and can include skin hypopigmentation and café-au-lait spots, skeletal anomalies like short stature, malformations of the upper and lower limbs, malformed or absent kidneys, malformations of genitalia, urinary and reproductive system including (predominantly) male infertility, gastrointestinal abnormalities, ophthalmic abnormalities, heart defects, abnormalities of the central nervous system, microcephaly, malformed ears, and hearing loss. In addition, around 10% of patients develop mild to moderate intellectual disability.

Similar to the majority of the above-mentioned genome instability syndromes, FA is mostly inherited in an autosomal-recessive manner, Meaning that the parents of affected individuals are usually unaffected heterozygous carriers, bearing the mutation on only a single allele. So far, biallelic mutations in 19 genes, termed *FANC(-)A* to *FANC(-)U*, have been shown to cause FA. However, two exceptions to this inheritance pattern exist. Namely, mutations affecting the *FANCB*, located on the X-chromosome, are inherited in an X-linked manner, therefore the female carriers do not develop FA-associated clinical signs and symptoms. In addition, heterozygous *de novo* mutations in *RAD51* have also been connected to FA, representing an autosomal-dominant mode of inheritance (4).

Here, we report a case of unusual presentation of FA with a fatal outcome.

## Background

**Figure 1:**
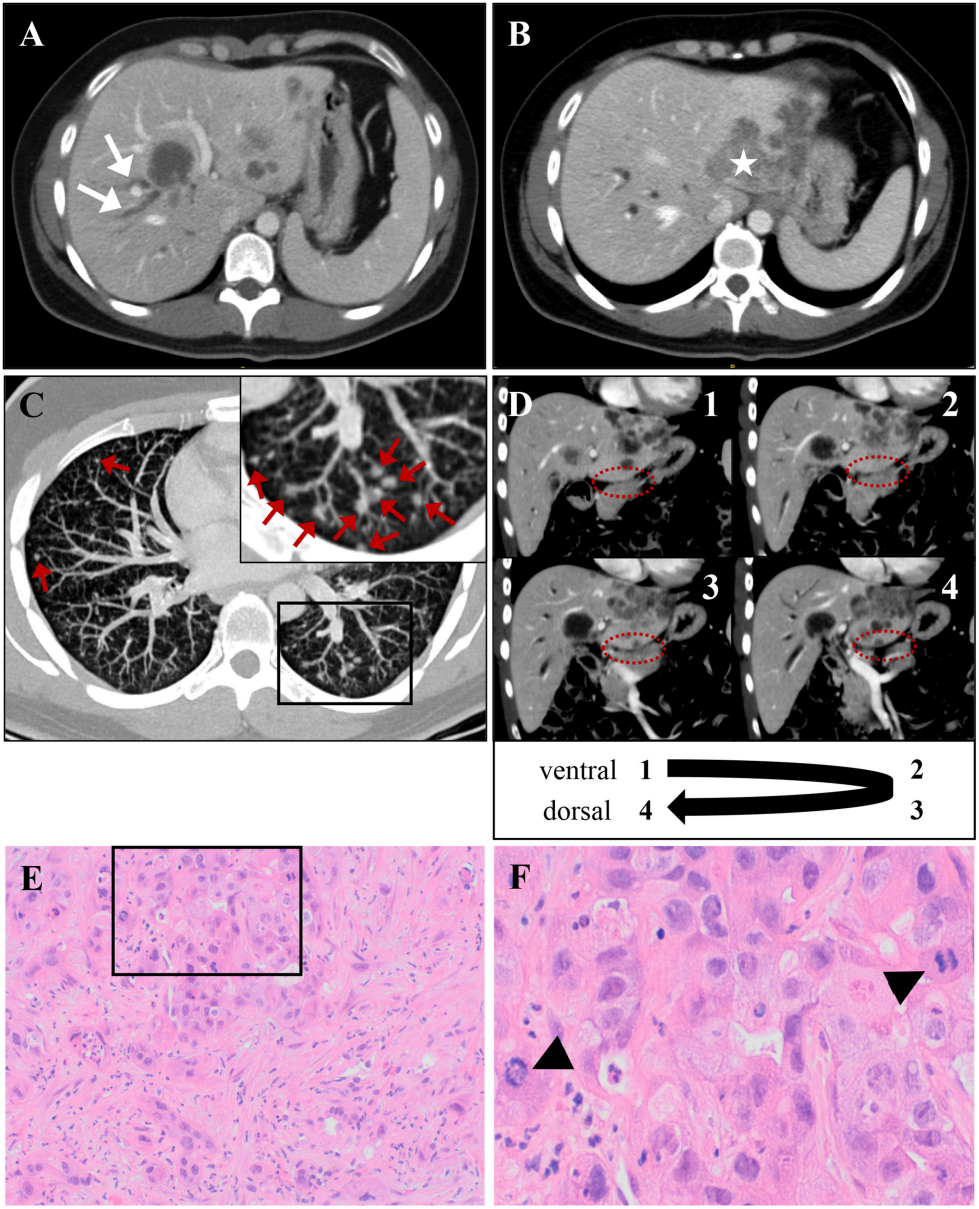
The patient's intrahepatic cholangiocarcinoma, which was classified as TNM: cT3, cN1, cM1, and stage IV tumor [according to AJCC/UICC staging criteria (6)]. **(A,B)** CT scan demonstrates multiple liver lesions, exemplary shown on two different axial layers. **(A)** Arrows indicate right-sided cholestasis, caused by a hypodense lesion in liver segments IV/VIII. **(B)** Asterisk marks a large, lobular-configured tumor mass in liver segment II, that dorsally crosses liver capsule. **(C)** A representative axial CT scan layer shows multiple bilateral lung noduli, interpreted as pulmonary metastases (indicated by red arrows). A framed image section is displayed as magnified inset in the top left corner of (C). A likely single spleen metastasis and enlargement of retroperitoneal lymph nodes are not shown. **(D)** The unaffected pancreas neighboring the infiltrated liver is depicted in four serial CT scan layers in coronal plane (consecutively numbered 1–4 from ventral to dorsal). The border between both organs (highlighted by dashed ovals) is not obviously violated by infiltrating tumor tissue, arguing against a primarily pancreatic origin of malignancy. **(E,F)** Histology from ultrasound-guided liver-biopsy. **(E)** H&E stain shows infiltrates of an adenocarcinoma with scattered duct formation and a desmoplastic stroma component. **(F)** High-power field from inset in (E) demonstrates frequent mitoses (indicated by arrow-heads). Immunohistochemistry (not shown) reveals positivity for CK7 and DPC4 and negativity for Hep Par 1, CK20, TTF1, CDX-2, and PSMA, consistent with a tumor of biliopancreatic origin.

A 33-year-old man of Turkish descent with stage IV intrahepatic cholangiocarcinoma according to the American Joint Committee on Cancer (AJCC)/Union for International Cancer Control (UICC) staging criteria (6) with distant metastases (e.g., to lungs; Figure 1) was admitted to our emergency room with a clinical and laboratory constellation consistent with sepsis. The patient had a quick Sequential [Sepsis-related] Organ Failure Assessment (qSOFA) score (7) of three points (tachypnea, altered mentation, systolic blood pressure 64 mmHg), a measured body temperature of 38.4°C (tympanic), acute kidney injury stage 2 according to Kidney Disease: Improving Global Outcome (KDIGO) criteria (8) (serum creatinine 2.1 mg/dl), an elevated lactate level (lactate 5.9 mmol/l, pH 7.36) and markedly elevated markers of inflammation [C-reactive protein (CRP) 199 mg/dl, procalcitonin (PCT) 26 μg/l]. The patient's complete blood count (CBC) showed severe pancytopenia [hemoglobin (Hgb) 7.5 mg/dl, hematocrit (Hct) 20.9%, white blood cells (WBC) 0.2 × 10^9^/l, platelets (PLT) 39 × 10^9^/l], raising concern about a neutropenic fever syndrome. The patient's history revealed, that he was on day 11 of his first cycle of a standard chemotherapeutic regime of gemcitabine (500 mg/m^2^; dose reduction of 50% due to bilirubinemia prior to treatment initiation) and cisplatin (25 mg/m^2^) for advanced cholangiocarcinoma (9), which he had received in an outpatient setting on days 1 and 8. Thus, a neutropenic fever grade 4 according to Common Terminology Criteria for Adverse Events (CTCAE, version 5) (10) was considered as an unexpected complication to this particular palliative chemotherapy regimen. An empiric broad-spectrum anti-infective therapy according to our institutional guidelines comprising the intravenous administration of meropenem, metronidazole, and vancomycin was immediately initiated. Additionally, packed red blood cells and granulocyte-colony stimulating factor (G-CSF) from the day of admission were given and the patient was transferred to intensive care unit (ICU) for initial management and monitoring.

Extensive diagnostic work-up for possible sites of infection included thorough physical examination, and repetitive blood and urine culture sampling. A computed tomography (CT) scan of chest and abdomen led to suspicion of neutropenic colitis. Stent placement therapy for a tumor-compression of the right hepatic duct (cf. Figure 1A) with moderate peripherical cholestasis was performed, but demonstrated no clear evidence for cholangitis. Bacteremia with vancomycin-resistant enterococcus species was detected over the course of hospital admission and addressed by appropriate adaption of antibiotic regimen (switch from vancomycin to linezolid). Moreover, empiric antifungal therapy using fluconazole was timely initiated. Transfusions of red blood cells and platelets were repeatedly performed. However, irrespective of therapeutic approach, the patient's clinical condition increasingly deteriorated with persisting signs of inflammation and no sustainable signs of bone marrow recovery until day 22 of chemotherapy cycle (Hgb 11.2 mg/dl, Hct 32.0%, WBC 0.2 × 10^9^/l, PLT 16 × 10^9^/l). Notwithstanding continuous G-CSF stimulation, not a single day with leukocytes >0.5 × 10^9^/l was recorded during hospital stay. Given uncontrolled infection in the setting of refractory aplasia and the context of a palliative tumor setting, intensive care treatment was withheld, and the patient deceased on day 25 of his first cycle of chemotherapy and 14 days after hospital admission, making for an unusual grade five toxicity of a first cycle of palliative chemotherapy for this patient.

## Phenotypic Features and Family History

Two days prior to death, the constellation of bone-marrow failure after exposure to a DNA crosslinking chemotherapeutic drug (cisplatin) and the presence of early-onset solid malignancy at the age of 33 years pointed to the possibility of an inherited DNA repair defect. A thorough evaluation of phenotypic features and past medical history identified several physical features suggestive of a hereditary syndrome. The patient displayed a short stature of 158 cm [1.8th percentile, −2.1 SD from average for Turkish men (11)] and had a solitary pelvic kidney representing a congenital kidney malformation diagnosed 7 years before. Additionally, his parents retrospectively reported a past surgery for a malformation of the left thumb at the age of 5 years and multiple surgeries for correction of bilateral ear canal malformations during childhood back in Turkey. A premature graying of the beard hair at the age of 18 years was also reported. However, the patient's CBC was never judged abnormal. It was completely normal 7 years before and only showed a moderate normocytic normochromic anemia prior to initiation of chemotherapy (Hgb 9.5 mg/dl, Hct 30.6%, WBC 9 × 10^9^/l, PLT 464 × 10^9^/l), which might also be explainable in the setting of advanced malignancy. The patient had a healthy 27-year-old brother of normal stature. The patient's parents and grandparents anamnestically displayed no phenotypic signs of hereditary syndromes. Besides, there was evidence in this patient's medical records for a long lasting unspecific chronic liver disease with undulating elevation of transaminases and cholestatic parameters for a period of ~5 years prior to diagnosis of cholangiocarcinoma, for which no clear cause had been established. A viral form of hepatitis, autoimmune biliary or liver diseases, hemochromatosis or secondary iron overload, gallstone disease, larger congenital malformations of the biliary tract as well as cirrhosis had been ruled out in previous hepatologic evaluations. The pathologist's interpretation of a liver biopsy, obtained 4.5 years prior to the diagnosis of cholangiocarcinoma, was toxic hepatitis, although a linked toxic agent could not be identified.

## Genetic Testing

**Figure 2:**
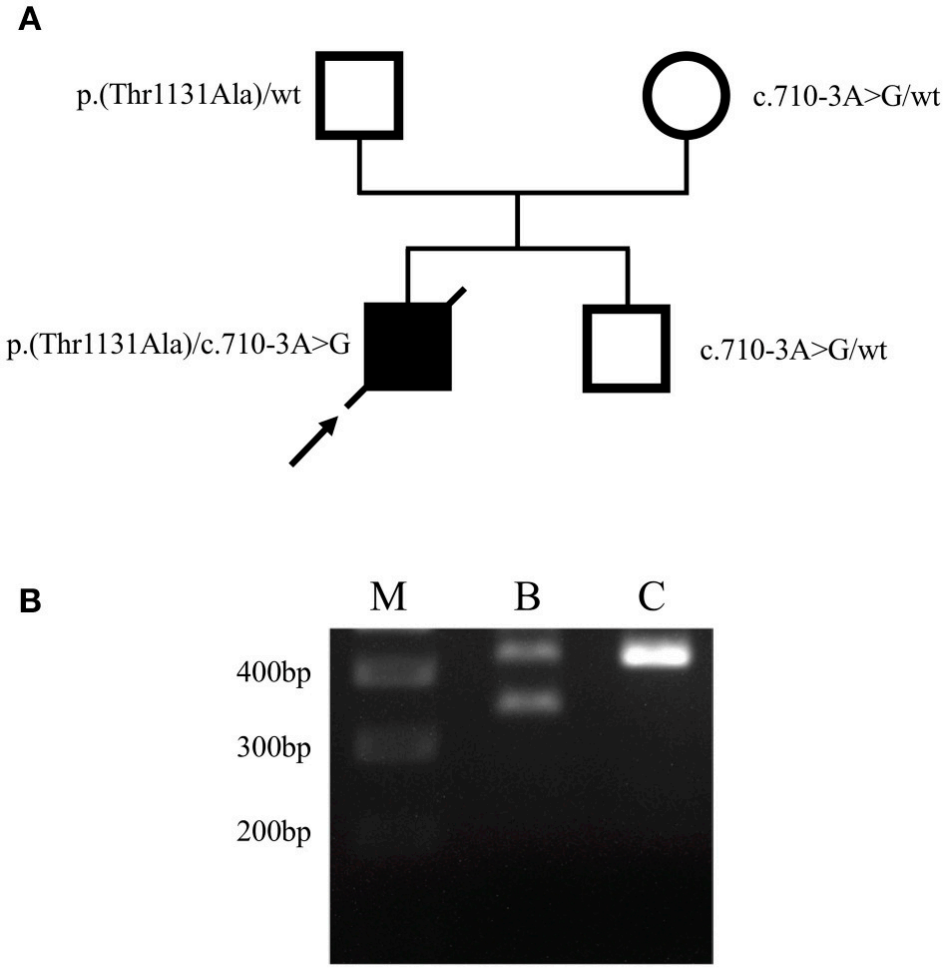
Identification of causative *FANCA* mutations. **(A)** Pedigree of the family. Filled and open symbols denote affected and healthy individuals, respectively; an arrow indicates the index patient, diagonal lines indicate deceased status. The mutation status is shown next to each symbol. **(B)** Agarose gel electrophoresis of *FANCA* RT-PCR products performed using lymphocyte-derived RNA of the patient's brother (B) who bears the heterozygous c.710-3A>G mutation, and a healthy individual (C), generated using primers F5′-AAGGCATTGTG AGCCTGCAAGA-3′ and R5′-ACAGGGCTGTGAGTGAGTATCTGA-3′. The 436-bp wild-type RT-PCR amplicon was amplified in both the patient's brother (B) and the control (C), using exon 8 flanking primers. A second, smaller PCR product of ~350 bp was only obtained in the patient's brother (B), corresponding to skipping of exon 8 (exon 8 contains 83 bp). The 400, 300, and 200 bp reference bands of the molecular marker (M) are indicated.

To unravel the putative genetic cause of patient's condition we performed whole-exome sequencing (WES) from DNA isolated from buccal swabs, as previously described (12). Buccal swab samples were collected 2 days prior to death (on day 12 after hospital admission). Bioinformatic filtering concentrated on rare variants within Mendelian cancer-risk DNA-repair genes, from the curated COSMIC germline cancer census gene set (v86; http://cancer.sanger.ac.uk/census). This analysis revealed two heterozygous variants in *FANCA*. Namely, a missense variant c.3391A>G, (p.Thr1131Ala), that had already been associated with FA (13–19), and a novel variant within the splice acceptor site of intron 7, c.710-3A>G. *In silico* prediction using NNSplice (20) indicated a disrupted splice acceptor site for this variant. Notably, a disrupting variant of the same splice acceptor (c.710-5T>C) had been previously described in a FA patient and was shown to induce an aberrant FANCA isoform by skipping of exon 8 (21). Segregation analysis with DNA samples of both healthy parents and his brother revealed the missense variant to be paternally inherited, whereas both the mother and his brother only bear the c.710-3A>G variant (Figure 2A), confirming the compound heterozygosity and an autosomal recessive inheritance. Further, RT-PCR analysis performed with RNA isolated from lymphocytes of the healthy brother as previously described (22) revealed that the c.710-3A>G variant causes skipping of exon 8 (Figure 2B), thus confirming its pathogenicity and allowing the post mortem diagnosis of FA in the index patient. Notably, primary dermal fibroblasts from a FA patient harboring the c.710-5T>C variant of *FANCA* (in compound heterozygosity with c.3558insG; FA-52 cells), that also causes skipping of exon 8 (21), demonstrated chromosomal instability after exposure to diepoxybutane (23). Furthermore, a previous study has shown that the c.3391A>G variant in *FANCA* significantly decreases FANCD2 monoubiquitination and the number of FANCD2 foci before and after mitomyocin C treatment (24), which is the critical event in the Fanconi/BRCA pathway for initiation of DNA repair (25). Taken together, the herein performed genetic analyses identified compound heterozygosity for two clearly pathogenic *FANCA* mutations thereby allowing the post-mortem clinical diagnosis of FA according to the published guidelines (4).

## Discussion

Fanconi Anemia is typically considered a pediatric disorder [median age at diagnosis 4.8–7.5 years (26, 27)] and this patient's phenotypic presentation was exceptional in two aspects. First, he never presented with hematologic abnormalities until initiation of systemic tumor therapy, although the majority of FA patients develop pancytopenia as a pathognomonic hallmark within the first decade of life, and the cumulative incidence of bone marrow failure is reported to be 90% by the age of 40 (28). Second, even though the patient was diagnosed with a relatively early-onset solid malignancy [median age at diagnosis for intrahepatic cholangiocarcinoma 67 years (29)], raising concern of an inherited condition, this is to our knowledge the first reported case of cholangiocarcinoma associated with FA so far. Notwithstanding, FA patients are at high risk for the development of myelodysplastic syndrome, acute myeloid leukemia and various solid malignancies especially head and neck squamous cell carcinomas (HNSCCs) (26, 30). FA patients are also prone to the occurrence of liver tumors (comprising hepatocellular carcinoma, hepatic adenoma, and hepatoblastoma) (31–34) However, formation of liver tumors is mainly observed in FA patients receiving androgen treatment (32) for palliation of bone marrow failure. Notably, the herein described patient was never on androgen treatment nor ever had an indication to be put on. Biliary tract tumors in FA patients have not been reported in literature (35). However, this patient had displayed blood value alterations non-specifically indicative of chronic liver disease years before the onset of tumor formation, which is a known phenotype in FA (33). Notably, chronic liver disease of various etiologies may contribute to the risk of cholangiocarcinoma formation in the general population (36) and might have facilitated cholangiocarcinoma formation in this patient with FA background. Moreover, the patient‘s premature hair graying, although not a validated feature of FA, is a common clinical sign of many genome instability disorders (12, 37). Thus, the finding of premature graying of the beard hair in the herein presented patient actually further expands the phenotypic spectrum of FA.

The patient's rapid demise most likely resulted from his particular treatment, particularly utilizing the gemcitabine/cisplatin chemotherapeutic regime. Thus, a timely awareness of underlying FA would have resulted in a different therapeutic approach with a putatively better outcome. Hypersensitivity to DNA crosslinking agents, including cisplatin (38–41), is one of the major features of FA (42), even utilized in diagnostic procedures, and fatal toxicity in FA patients exposed to cisplatin has already been documented (43–45). The gemcitabine plus cisplatin combination therapy remains the mainstay for the treatment of advanced cholangiocarcinoma and allow for a median overall survival of ~12 months (9, 46, 47). By choosing the gemcitabine combination partner cisplatin, the actual survival of this patient was 24 days from start of therapy due to fatal myelotoxicity. Noteworthy, a speculative combination with either capecitabine or S-1 (which is available in Germany), that demonstrated comparable efficacy to cisplatin in sporadic cholangiocarcinoma (46, 47), although head-to-head comparison data of gemcitabine-combinations is missing, might have been better tolerated. Even a conservative approach with gemcitabine monotherapy allows for a median overall survival of ~7 months (9) in sporadic bile duct cancer. Gemcitabine, capecitabine and tegafur (antineoplastic component of S-1) all belong to the class of pyrimidine analogs which exert cytotoxicity by inhibiting DNA replication, a mechanism of action thought to be of better tolerability in FA patients compared to direct DNA damage by crosslinking agents (48, 49). Genotoxic agents of this group (e.g., fludarabine, cytarabine) were well-tolerated in stem cell transplantation conditioning regimes for pediatric FA patients treated for myeloid malignancies (48, 50).

This is not the first report of delayed diagnosis of FA, being diagnosed only after unforeseen toxicity of DNA crosslinking agents in a primarily presumed sporadic-onset malignancy (44, 51). Thus, FA should be ruled out in early-onset malignancies, especially, if a typically FA-associated malignancy is diagnosed. However, the onset of a typically non-FA associated tumor type, such as cholangiocarcinoma does not rule out atypical presentation of FA, as this case demonstrated. Thus, the possibility of an underlying hereditary syndrome in cases manifesting as early-onset malignancies should be considered prior to the start of treatment. Notably, excessive toxicity following antineoplastic therapy is not solely attributable to FA, but has been observed in a broad range of genome instability syndromes, including Nijmegen breakage syndrome (52), Xeroderma pigmentosum (53), Bloom syndrome (54), Werner syndrome (55), Ataxia-telangiectasia (56), and Ruijs–Aalfs syndrome (37). Although all of these conditions are exceedingly rare and many of these patients become diagnosed early in life due to the multisystem nature of these disorders, some might actually lack the exact diagnosis prior to onset of malignancy formation. A further diagnostic burden represents the fact that although all genome instability disorders can result in multi-organ symptoms, the primarily affected organs, clinical presentations, and courses are highly variable. Moreover, based on the underlying genetic cause and the affected genome maintenance system, resulting DNA lesions and hypersensitivity toward genotoxic agents highly differ between these syndromes (Table 1). Thus, the precise elucidation of the underlying genetic cause represents the prerequisite for the selection of optimal antineoplastic therapy in genome instability disorders. Modern genetic analyses, e.g., whole-genome sequencing (WGS) by next-generation sequencing (NGS) technologies, covering the complete human genome, can utilize successful differential diagnosis of a genetic disorder within 50-h (57). Therefore, it is theoretically possible to perform those analyses without accepting an unfavorable delay of treatment initiation in tumor patients. However, these tests can also identify variants of unknown significance (VUS), whose pathogenicity can sometimes not be deduced without time consuming follow-up analyses. A further burden, at least in Germany, is the fact that the health insurance covers only WES, which compared to WGS does not properly cover the whole genome and even results in the poorer coverage of coding regions (exons), not to mention that it takes considerably longer, up to 2 weeks. It is further worth noting that NGS-based testing of the tumor sample first, would have also likely identified two heterozygous FANCA mutations. Misinterpretation of this finding, which is not completely excluded without pairing to normal tissue, could have potentially led to misinterpretation with even recommending a cisplatin-based therapy in this patient as the onset of somatic Fanconi/BRCA pathway defects is a feature of some cisplatin-sensitive tumors (58, 59). For experienced investigators, such a fault is unlikely to make, though. Indeed, the vast majority of early-onset cancer patients, which are either non-syndromic patients or have a negative family history, receive genetic analyses of normal tissue only after a mutation in a gene known to be associated with germline cancer predisposition is first identified in the tumor sample (60). However, the targeted re-analysis of the normal sample takes additional time, either resulting in treatment delay or start of an inappropriate therapy. Therefore, we suggest that in cases of early-onset cancer a paired tumor and normal NGS-based analysis should be performed, which additionally reduces the number of identified VUS (60).

**Table 1:** Comparison of typical features of various genome instability syndromes.

**Genome instability syndrome**	**Affected gene(s)**	**Clinical characteristics**	**Known cancer predisposition (not exhaustive)**	**Affected genome maintenance system**	**Agents to avoid or to use with special caution**
Fanconi anemia	Twenty-one genes (*FANCA* to *FANCU*)	Bone marrow failure, short stature, abnormal skin pigmentation, skeletal malformations, ophthalmic, and genitourinary tract anomalies	Acute myeloid leukemia, myelodysplastic syndrome, head and neck squamous cell carcinomas	Interstrand crosslink repair	Alkylating agents and platinum-based antineoplastic drugs, e.g., cisplatin, mitomycin c
Nijmegen breakage syndrome	*NBN*	Microcephaly, intrauterine growth retardation and short stature, recurrent infections, intellectual disability	T-cell and B-cell lymphomas, medulloblastoma, glioma, rhabdomyosarcoma	Double-strand break repair by homologous recombination	Ionizing radiation, various chemotherapeutic agents, e.g., etoposide, bleomycin, mitomycin c
Xeroderma pigmentosum	Nine genes (*DDB2, ERCC1-5 POLH, XPA*, and *XPC*)	Sun sensitivity, photophobia, keratitis, microcephaly, hearing loss, neuropathy, progressive cognitive impairment	Basal-cell carcinoma, squamous cell skin cancer, melanoma	Nucleotide excision repair	UV exposure, DNA adducts, e.g., cisplatin
Bloom syndrome	*BLM*	Intrauterine growth retardation and short stature, sun sensitivity, lipodystrophy, frequent infections, diabetes mellitus	Leukemia, lymphoma, squamous cell skin cancer, various solid tumors	Double-strand break repair by homologous recombination, DNA replication	UV exposure, ionizing radiation, various chemotherapeutic agents
Werner syndrome	*WRN*	Short stature, loss and graying of hair, scleroderma-like skin changes, bilateral cataracts, diabetes mellitus, hypogonadism, skin ulcers, osteoporosis, atherosclerosis, myocardial infarction	Soft-tissue sarcomas, osteosarcoma, thyroid cancer	Telomere maintenance and DNA replication	Various chemotherapeutic agents, e.g., cisplatin, mitomycin c
Ataxia-telangiectasia	*ATM*	Progressive cerebellar ataxia, oculomotor apraxia, choreoathetosis, telangiectasias of the conjunctivae, immunodeficiency, frequent infections	Acute lymphoblastic leukemia of T-cell origin, B-cell lymphomas, ovarian cancer, gastric cancer, melanoma, leiomyoma, sarcomas	Double-strand break repair by homologous recombination	Ionizing radiation
Ruijs–Aalfs syndrome	*SPRTN*	Cataracts, graying of hair, short stature, muscular atrophy, lipodystrophy, micrognathia	Hepatocellular carcinoma	DNA-protein crosslink repair	Alkylating agents and platinum-based antineoplastic drugs, e.g., mitomycin c, cisplatin

## Concluding Remarks

One major aspect of precision medicine in oncology is the identification of patients with germline cancer predisposing mutations, in order to optimize the initial therapy. The latter is further highlighted by the herein presented patient with normal hematologic values and a non-classically FA-associated early-onset cancer followed by fatal outcome due to therapy-induced myelotoxicity. In order to identify such patients, the clinical team should perform an in-depth investigation of patient's family history and previous medical records, followed by rapid and appropriate genetic analyses, in order to pinpoint or rule out a possibly underlying hereditary genome instability syndrome and to facilitate a rational treatment strategy in early-onset malignancies. However, it seems a vain attempt to derive a solid guideline for this rare clinical problem mostly grounded on experiences from sporadic case reports. Nevertheless, we propose a multidisciplinary management for any patient with an early-onset cancer (relatively with respect to patient's risk profile and epidemiologic data for the respective tumor entity). Those who additionally display at least subtle phenotypical features suggestive of a hereditary syndrome should be considered for paired tumor and normal NGS-based genomic testing.

## Ethics Statement

Collection of biological samples and genetic analyses were conducted following written informed consent from studied individuals or his legal representatives. In addition, written informed consent for the publication of this case was obtained from the family of the deceased patient. The study was performed in accordance with the Declaration of Helsinki protocols.

## Author Contributions

NE, SS, and DL designed the study. NE and DL prepared the manuscript. NE is responsible for clinical information gathering. NE and DL are responsible for sample collection and consent. US assisted with histology. CF, CB, CK, and DL supervised the study and data analysis.

### Conflict of Interest Statement

The authors declare that the research was conducted in the absence of any commercial or financial relationships that could be construed as a potential conflict of interest.
